# Supplementary material for: Convergent eusocial evolution is based on a shared reproductive groundplan plus lineage-specific plastic genes
Source: Nat Commun. 2019 Jun 14;10:2651. doi: 10.1038/s41467-019-10546-w (PMC6570765; doi:10.1038/s41467-019-10546-w)
Supplement: Supplementary file 1 — Supplementary Information [file 41467_2019_10546_MOESM1_ESM.pdf]

Supplementary Information

Convergent eusocial evolution is based on a shared reproductive groundplan plus lineage-specific plastic genes

Warner et al.

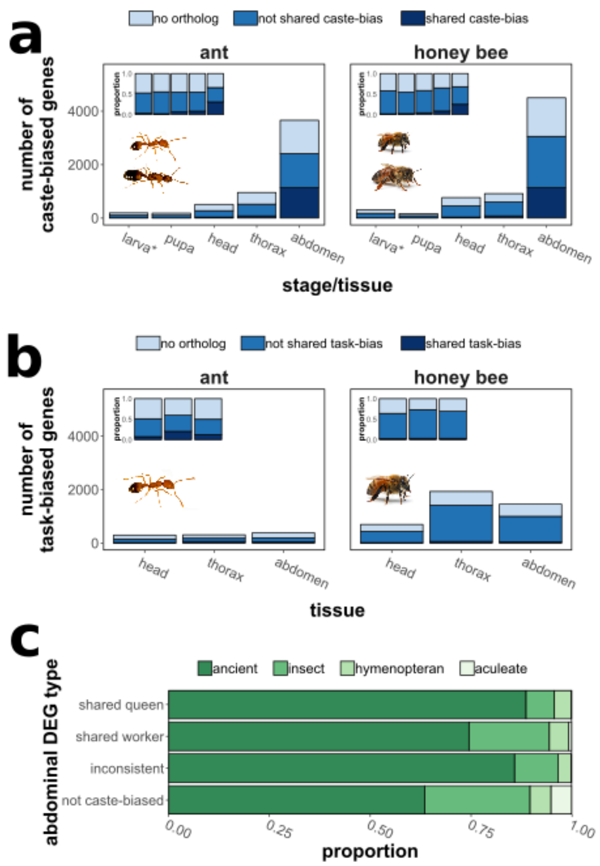

Supplementary Figure 1. Patterns of caste-biased expression in pharaoh ants and honey bees.
Number of differentially expressed genes (FDR < 0.05) between a) queens and workers and b) nurses and foragers at each developmental stage or tissue in ants (left) and honey bees (right). "Head", "thorax", and "abdomen" refer to body segments of adults, while "pupa" and "larva" refer to whole bodies. "No ortholog" refers to genes for which no 1:1 ortholog exists (either due to apparent duplication or complete lack or orthology), "not shared caste/task bias" refers to genes for which 1:1 orthologs can be identified but are only differentially expressed in one species, and "shared caste/task" bias refers to genes for which 1:1 orthologs are differentially expressed in both species. Insets show the proportion of each category of gene out of all differentially expressed genes at that stage or tissue. c) Proportion of abdominal DEGs by estimated evolutionary age (shading). "Shared queen/worker" indicates genes upregulated in queen or workers of both species. Source data are provided as a Source Data file.
*: the category "larva" represents differential expression across larvae of all stages for which caste can be identified (second to fifth larval stage).

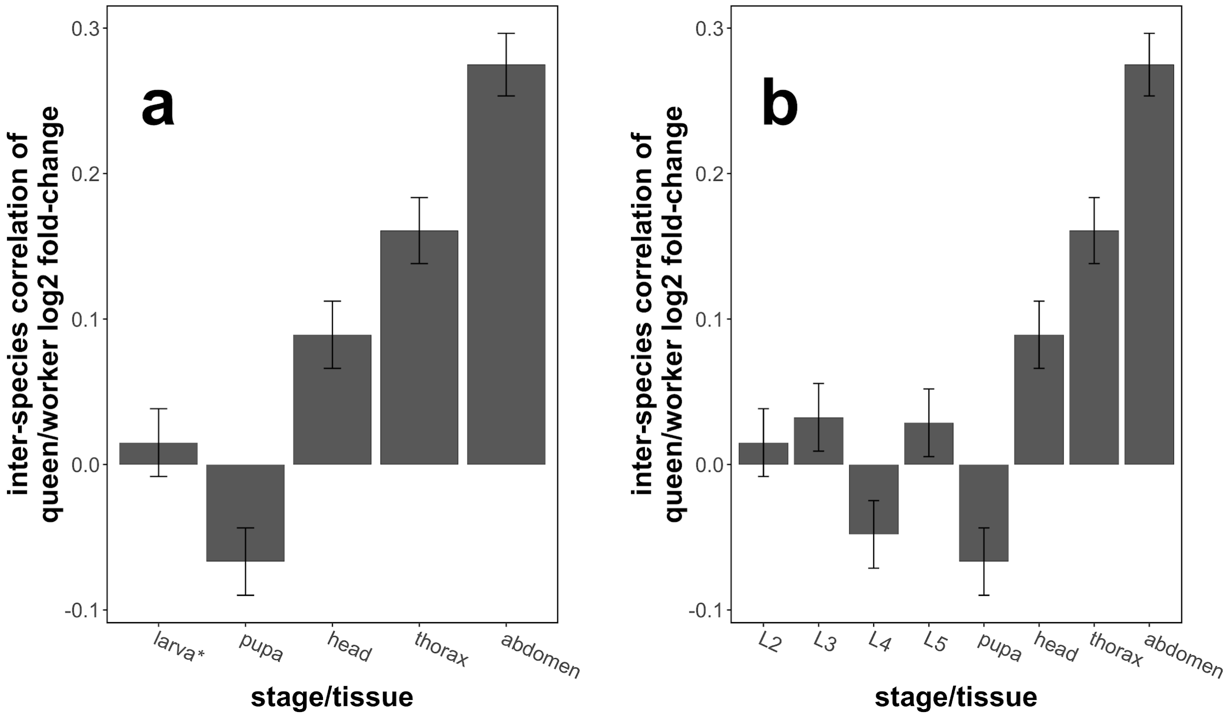

Supplementary Figure 2. Correlation of gene-wise $\log_2$ fold-change between queens and workers in ants and honey bees.

Pearson correlation of $\log_2$ fold-change between queens and workers as measured at each stage or tissue in *M. pharaonis* and *A. mellifera* for each 1:1 ortholog (N = 7640). Error bars indicate Pearson correlation 95% confidence intervals. In (a), the category "larva*" represents differential expression across larval stages, while in (b) each larval stage (L2-L5) is plotted individually. Source data are provided as a Source Data file.

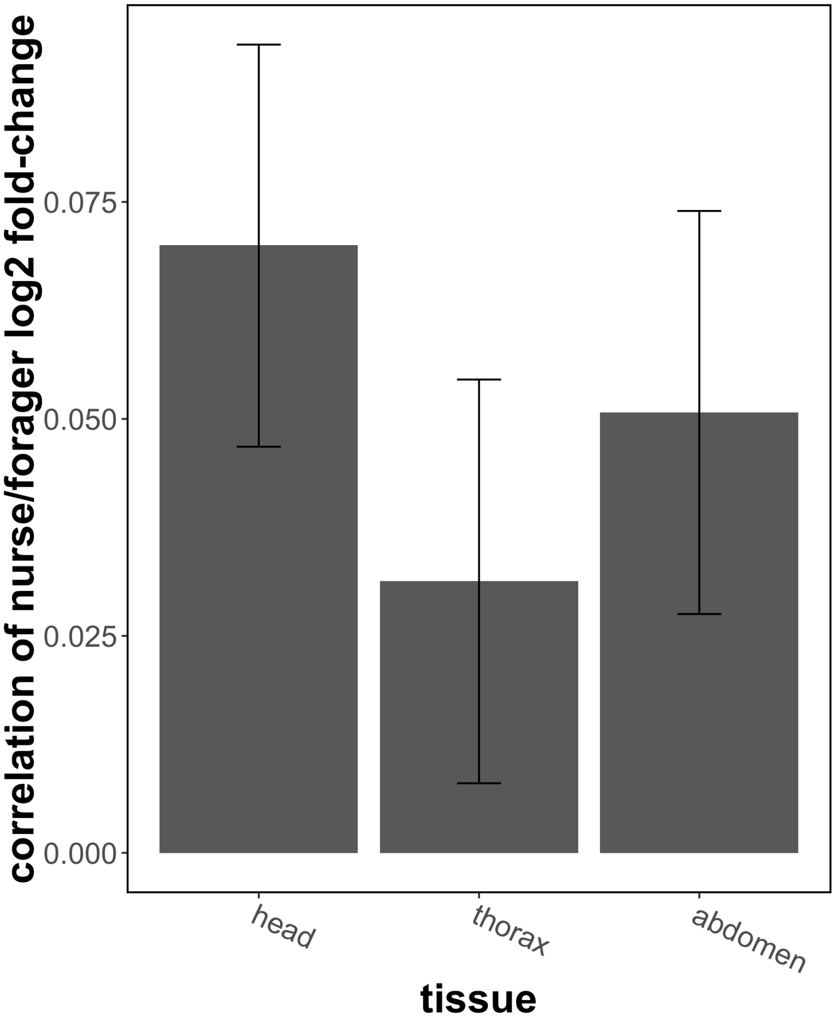

Supplementary Figure 3. Correlation of gene-wise log_2 fold-change between nurses and foragers in ants and honey bees.

Pearson correlation of log-fold change between nurses and foragers as measured in each tissue in *M. pharaonis* and *A. mellifera* for each 1:1 ortholog (N = 7640). Error bars indicate Pearson correlation 95% confidence intervals. Source data are provided as a Source Data file.

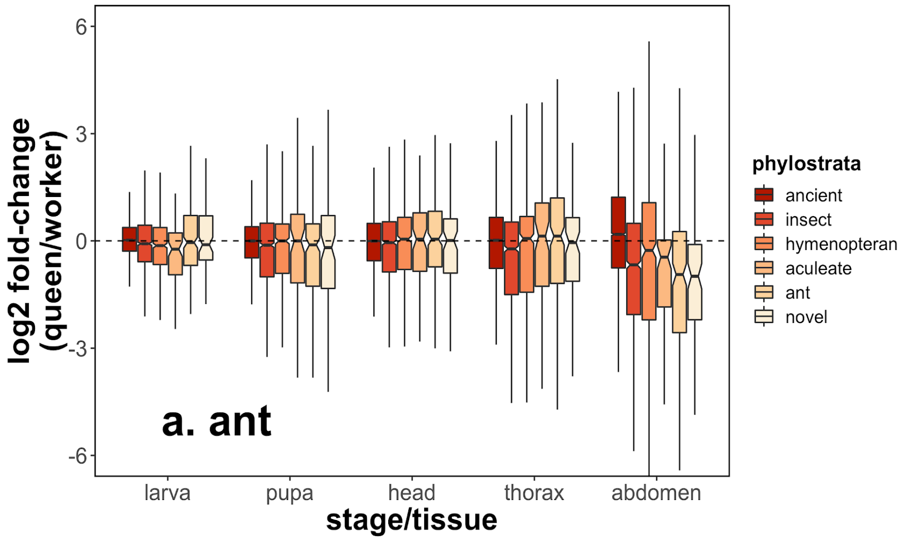

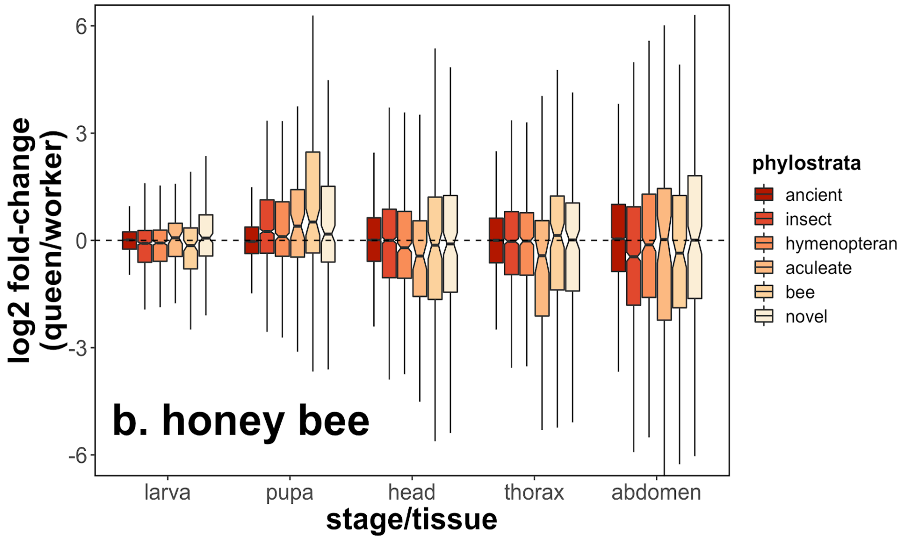

Supplementary Figure 4. Log$_2$ fold-change between queens and workers across phylostrata. Log$_2$ fold-change at each stage/tissue in each phylostrata category. Positive values indicate higher expression in queens compared to workers. Log$_2$ fold-change has been adjusted relative to the median value at that stage/tissue, in order to compare across tests. "Ancient" genes indicate any genes shared beyond insects (i.e. with vertebrates). Log$_2$ fold-change varies according to phylostrata for every stage/tissue for each species (LM; LRT; $P < 0.001$). In each boxplot, the middle line represents median values, outer edges of boxplot represent upper and lower quartiles, and whiskers represent a deviation of 1.5*(interquartile range) from the upper and lower quartiles. Source data are provided as a Source Data file.

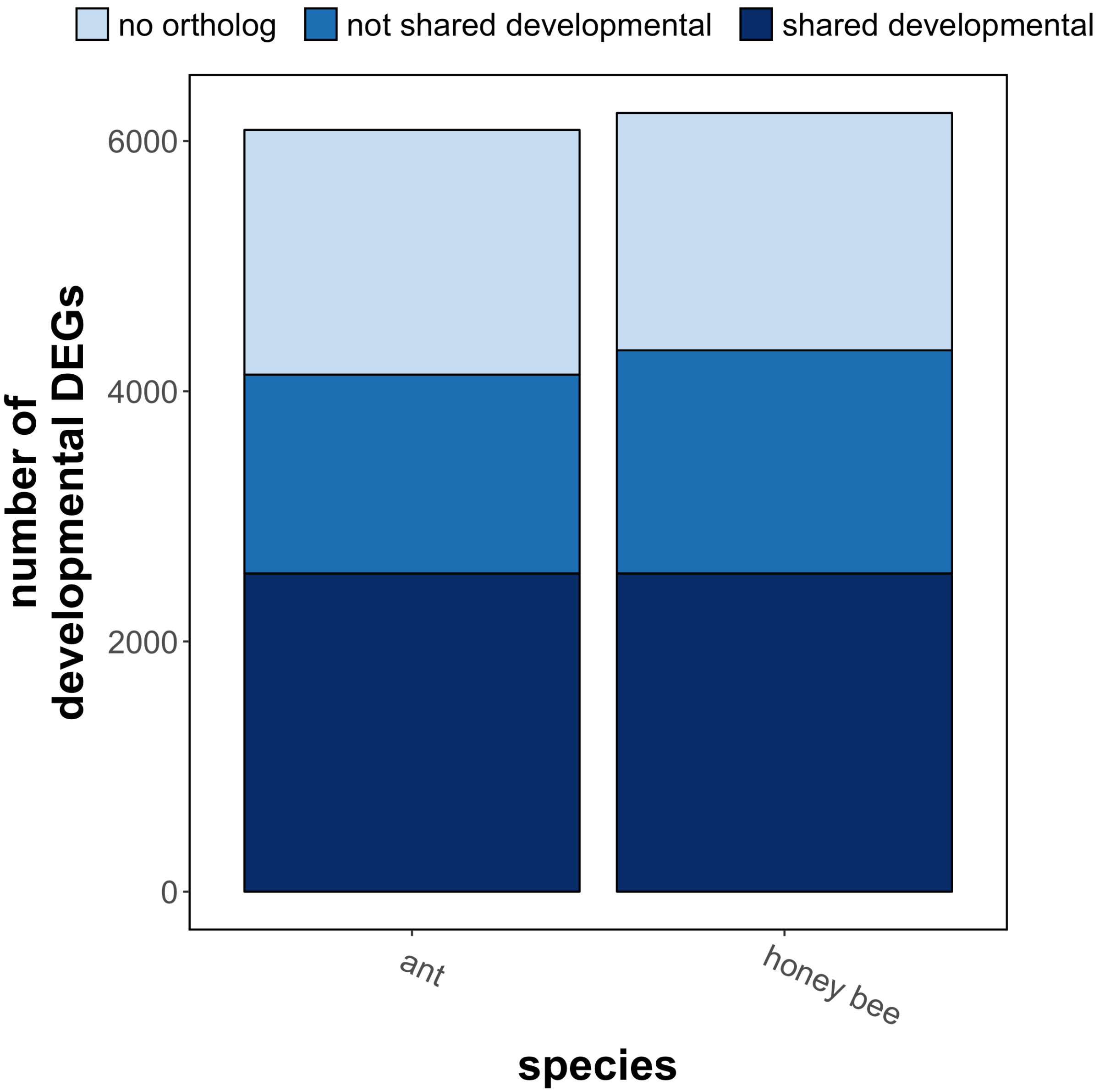

Supplementary Figure 5. Overlap of developmentally-biased genes in ants and honey bees. Number of developmental differentially expressed genes in ants and in honey bees. “No ortholog” refers to genes for which no 1:1 ortholog exists (either due to apparent duplication or complete lack or orthology), “not shared developmental” refers to genes for which 1:1 orthologs can be identified but are only differentially expressed between developmental stages in one species, and “shared developmental” refers to genes for which 1:1 orthologs are differentially expressed between embryonic and larval developmental stages in both species. Source data are provided as a Source Data file.

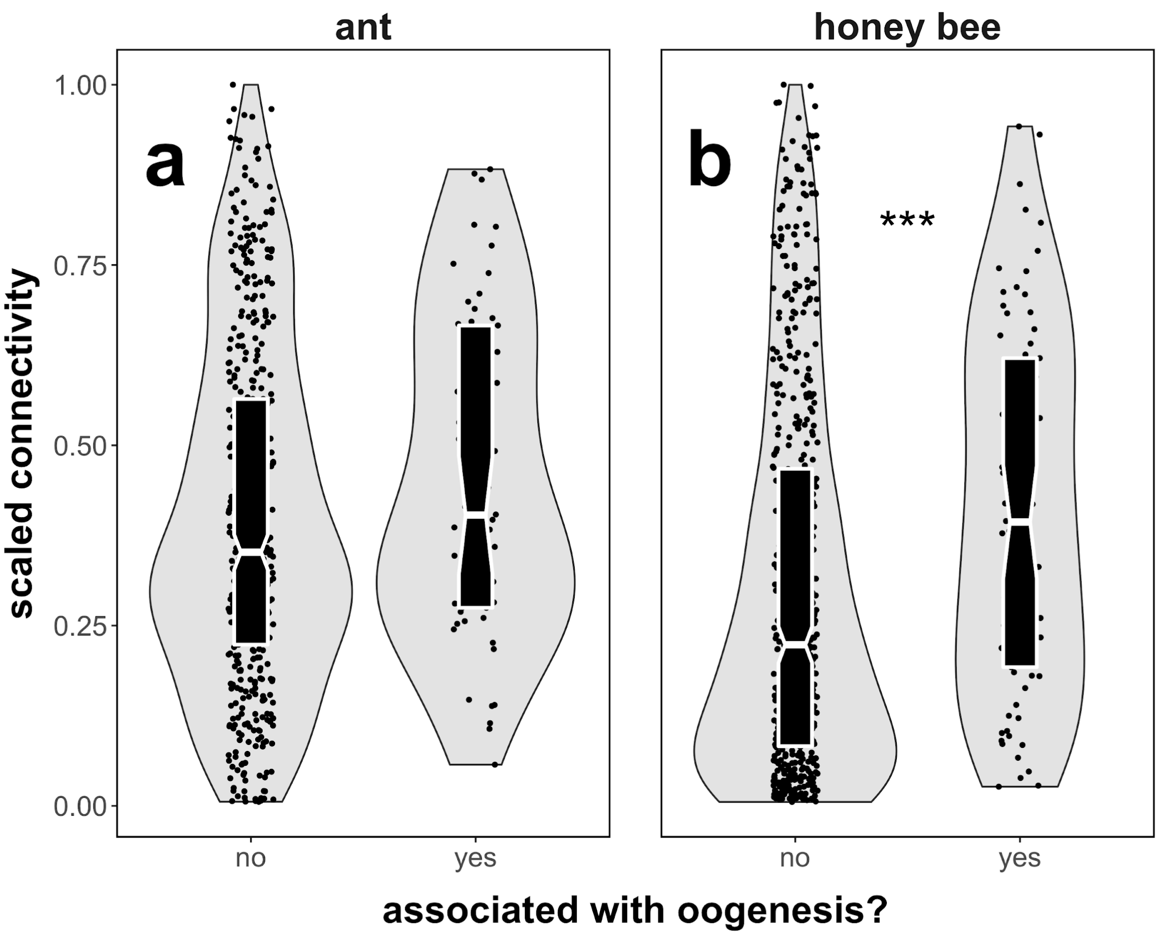

Supplementary Figure 6. Genes associated with oogenesisis are more highly connected within queen abdominal modules.

Orthologs of genes associated with oogenesis in *D. melanogaster* are more highly connected within queen abdominal modules in honey bees (*** = P < 0.001; Wilcoxon test) though not in ants (P = 0.114). N = 542 (ants), 649 (honey bees).). In each boxplot, the middle line represents median values, outer edges of boxplot represent upper and lower quartiles, and whiskers represent a deviation of 1.5*(interquartile range) from the upper and lower quartiles. Source data are provided as a Source Data file.

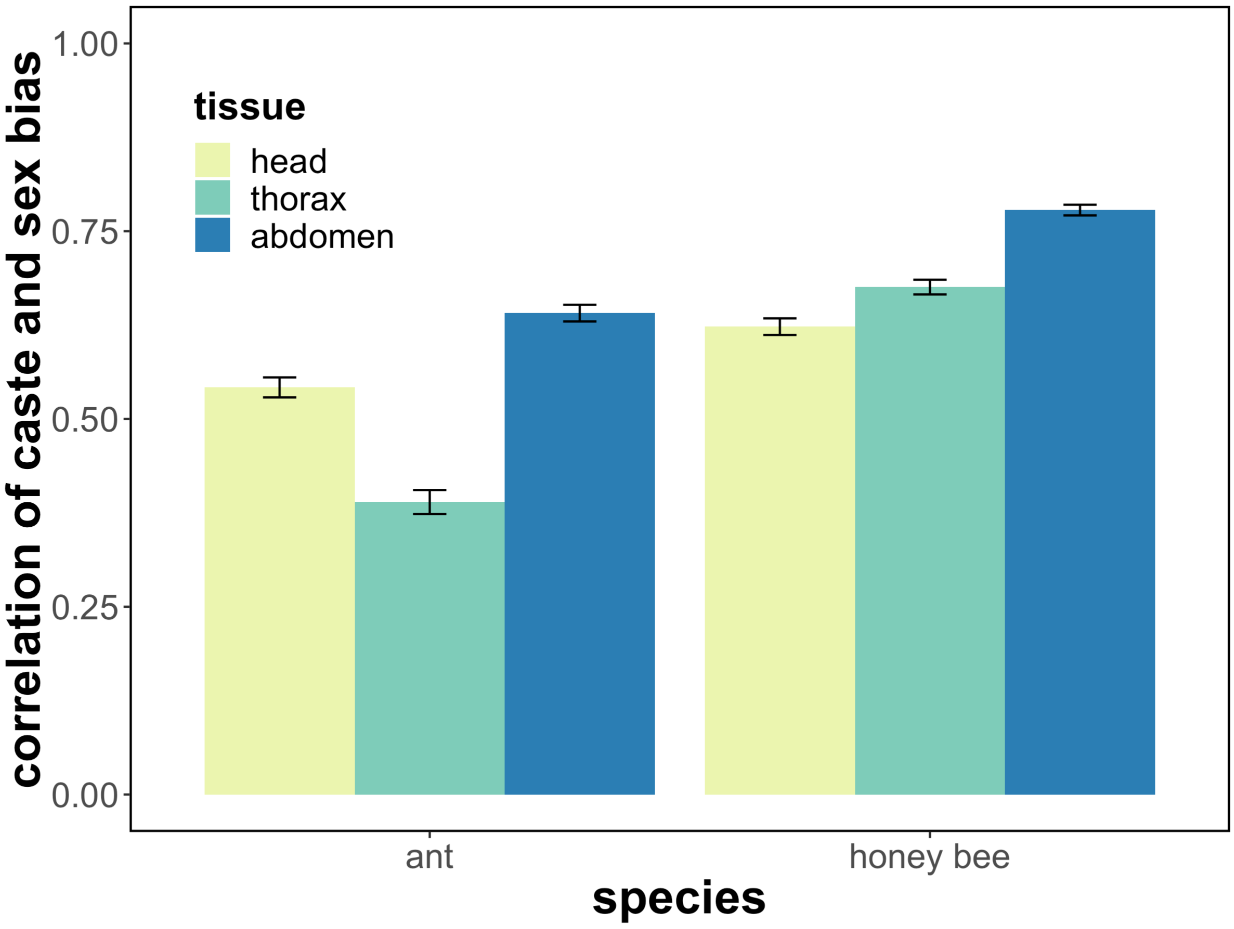

Supplementary Figure 7. Caste-biased expression is correlated to sex-biased expression. Pearson correlation of caste (queen/worker) and sex (queen/male) expression bias in ants and honey bees. Error bars represent Pearson correlation 95% confidence intervals. Correlations are significant in all cases (Pearson correlation; $P < 0.001$), but abdominal correlations are strongest. Source data are provided as a Source Data file.

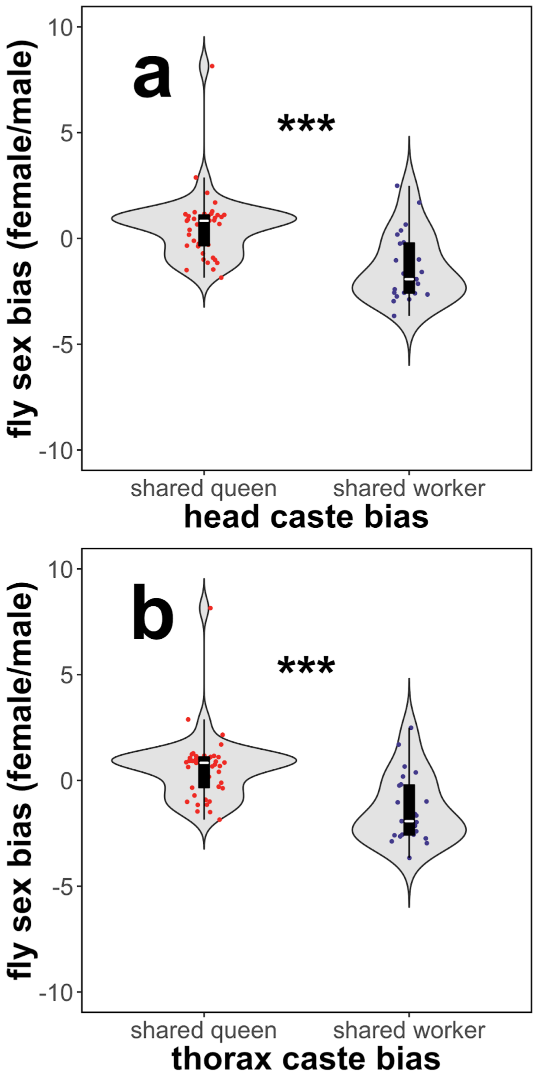

Supplementary Figure 8. Social insect queen-biased genes tend to be female-biased in *D. melanogaster*.

Shared queen-biased DEGs tend to be female-biased in *D. melanogaster* while shared worker-biased DEGs tend to be male-biased in *D. melanogaster* (likely reflecting down-regulation in females) in both a) head and b) thoracic tissues.). In each boxplot, the middle line represents median values, outer edges of boxplot represent upper and lower quartiles, and whiskers represent a deviation of 1.5*(interquartile range) from the upper and lower quartiles. Source data are provided as a Source Data file.

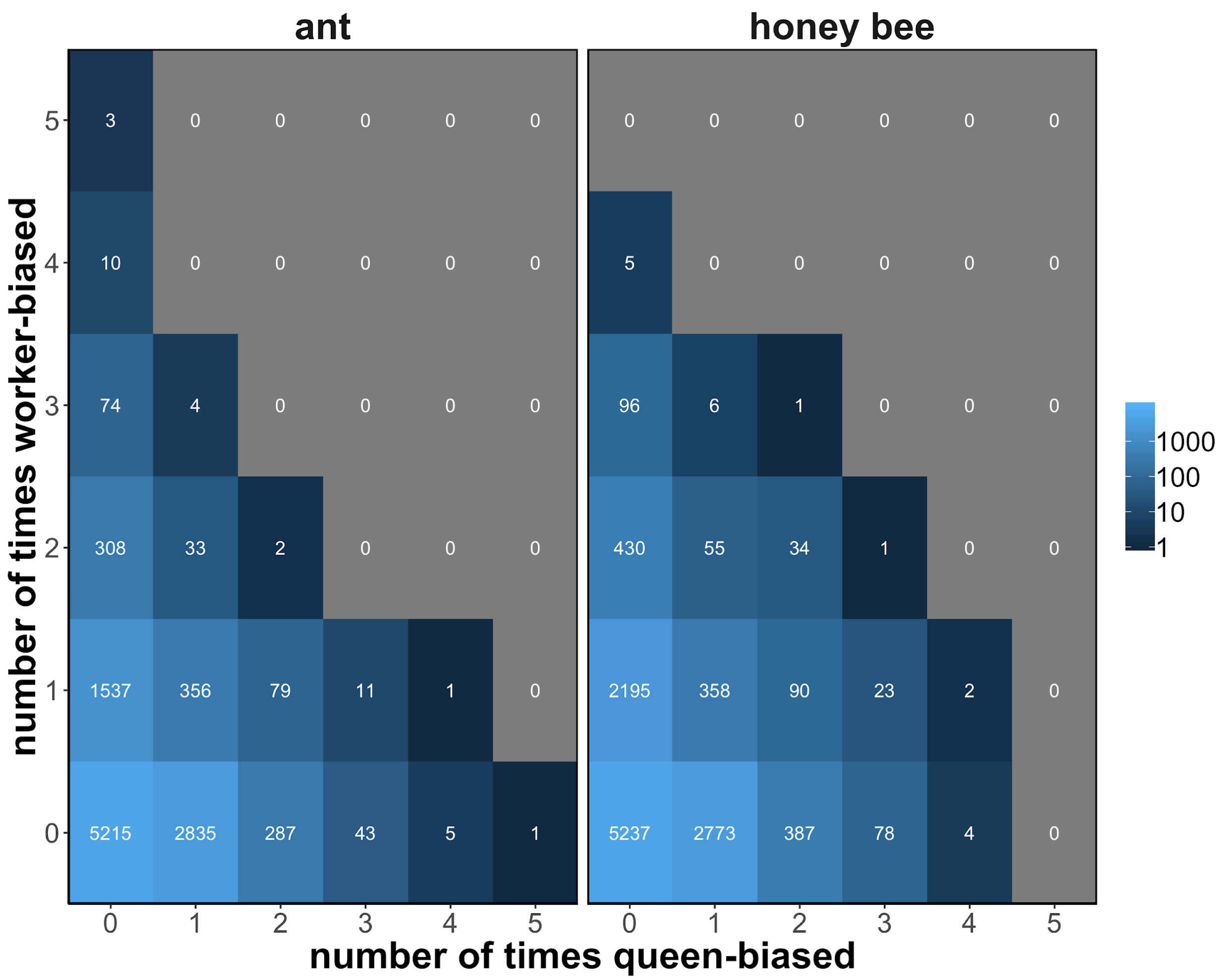

Supplementary Figure 9. Distribution of the number of times genes exhibit biased expression towards each caste.
Number of times each gene is upregulated in queen and workers across all comparisons (larva, pupa, and adult head, thorax, and abdomen). Color brightness is logarithmically proportional to the number of genes in each cell. N = 10804 genes for ants, N = 11775 genes for honey bees. Source data are provided as a Source Data file.

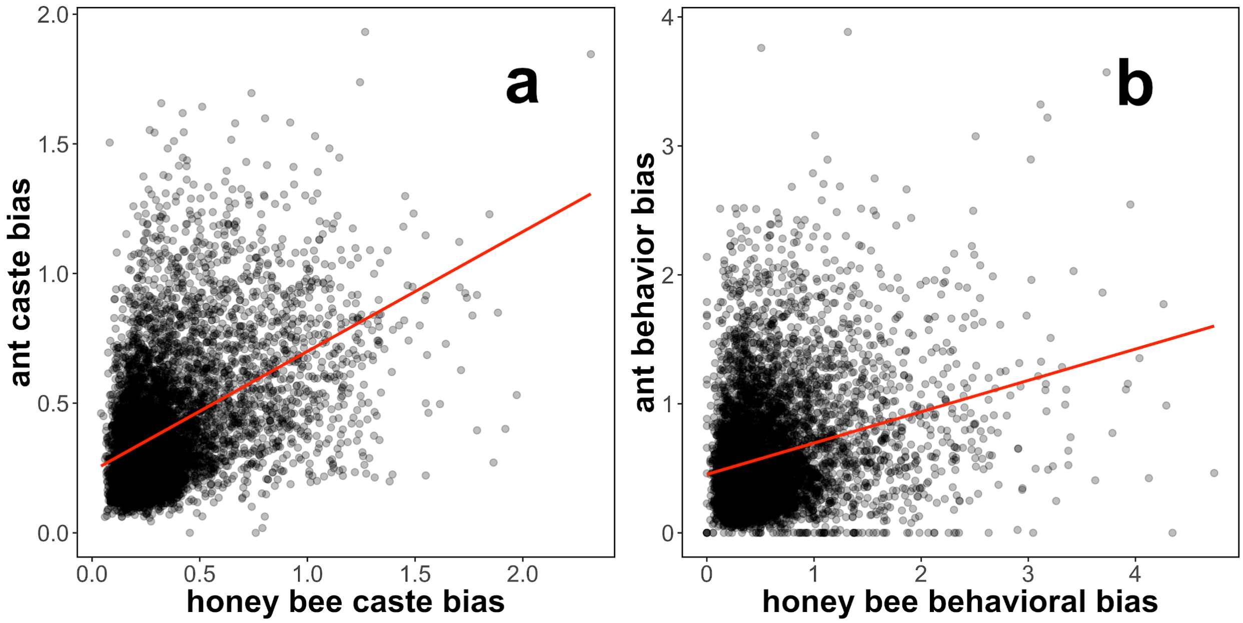

Supplementary Figure 10. Caste and behavior bias are correlated between species. Overall caste bias (a) and overall behavior bias (b) is correlated between ants and honey bees. "Overall" bias refers to the Euclidean distance of all $\log_2$ fold-change values (queens/worker for caste, nurses/foragers for behavior). The red line is the trendline of a linear model; Spearman correlation $P < 0.001$ in all cases. Source data are provided as a Source Data file.

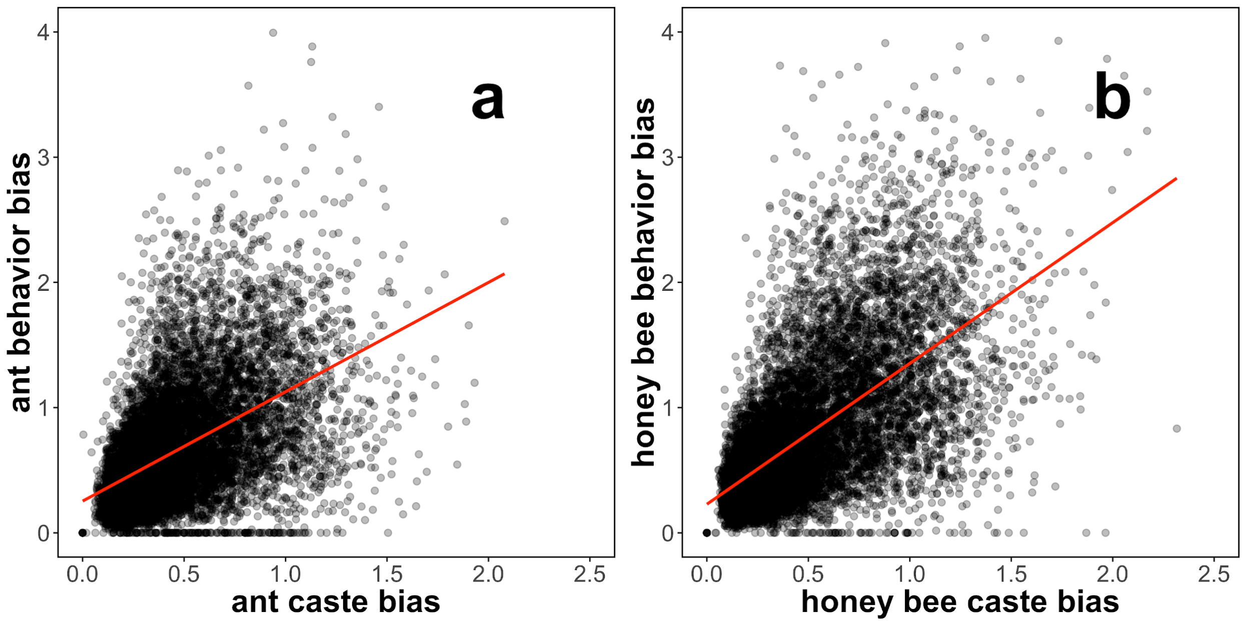

Supplementary Figure 11. Caste bias is correlated to behavior bias.
Overall caste bias and overall behavior bias were correlated within a) ants and b) honey bees. "Overall" bias refers to the Euclidean distance of all $\log_2$ fold-change values (queens/worker for caste, nurses/foragers for behavior). The red line is the trendline of a linear model; Spearman correlation $P < 0.001$ in all cases. Source data are provided as a Source Data file.

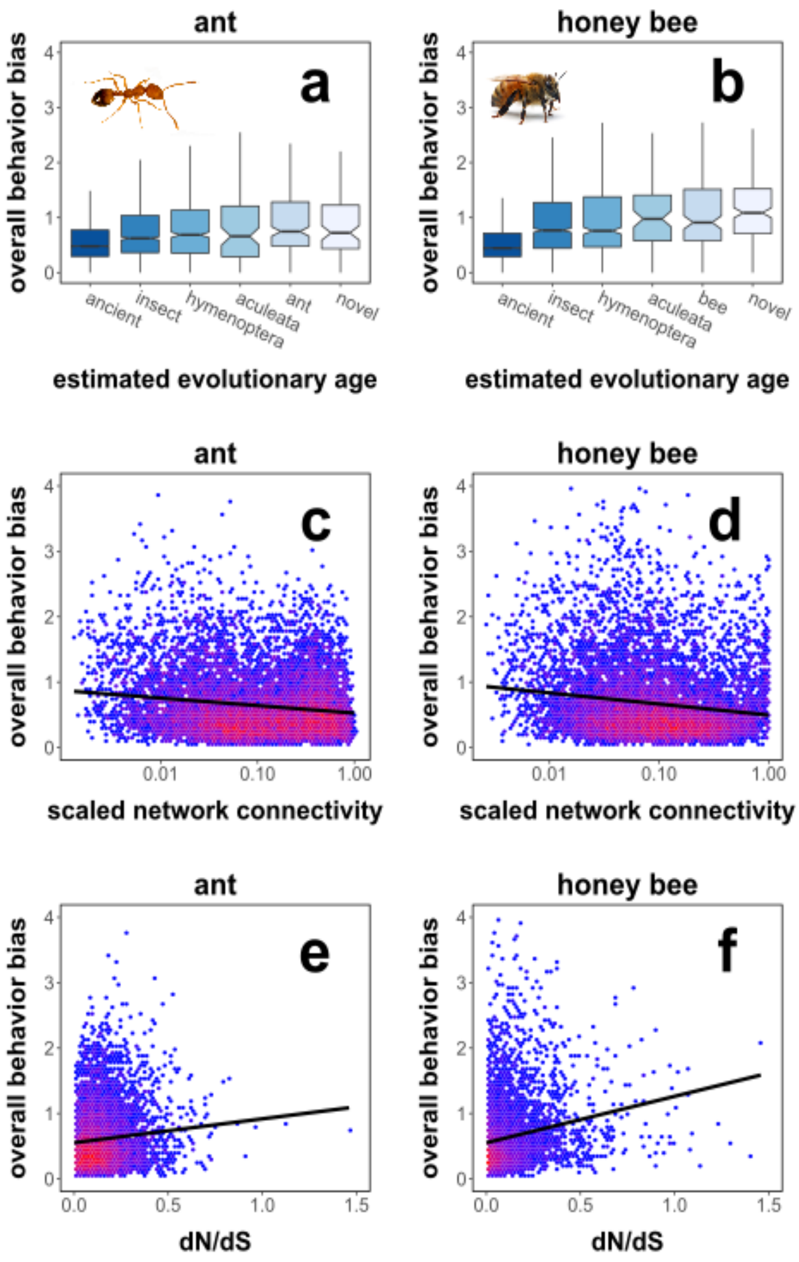

Supplementary Figure 12. Evolutionary and network features of behavior-biased genes. Genes that exhibit more behavior bias across tissues have younger estimated evolutionary ages (a,b) and tend to be loosely connected (c,d; Spearman correlation; ant: rho = -0.099, $P < 0.001$; honey bee: rho = -0.157, $P < 0.001$) and rapidly evolving (e,f; Spearman correlation; ant: rho = 0.079, $P < 0.001$; honey bee: rho = 0.226, $P < 0.001$). "Overall behavior bias" combines nurse forager $\log_2$ fold-change values across all adult body segments. Connectivity is calculated using all samples and genes and scaled proportionally to the highest value.). In each boxplot, the middle line represents median values, outer edges of boxplot represent upper and lower quartiles, and whiskers represent a deviation of 1.5*(interquartile range) from the upper and lower quartiles. Source data are provided as a Source Data file.

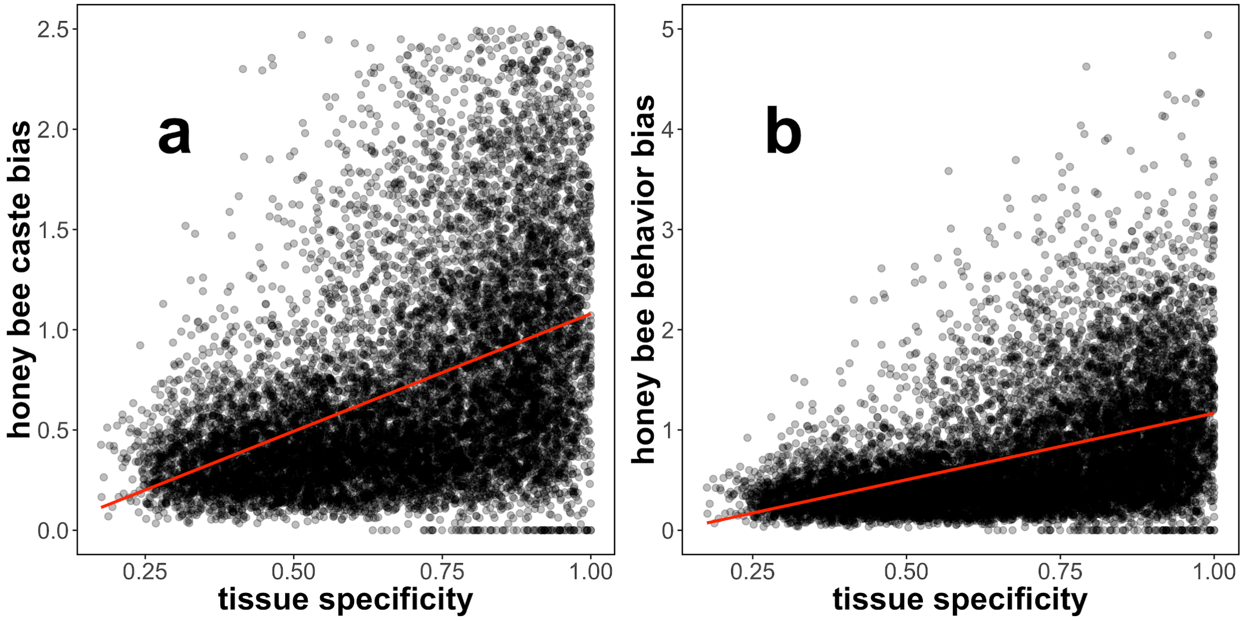

Supplementary Figure 13. Caste and behavior bias are correlated to tissue specificity in honey bees.

Genes exhibiting more behavior bias tend to be tissue-specific. There was a positive correlation (Spearman correlation, $P < 0.001$ in each case) between caste/behavior bias and tissue specificity, where tissue specificity (τ) is estimated using data from 12 honey bee tissues. $\tau = 1$ indicates a genes is expressed in only one tissue, while lower values indicate genes are more ubiquitously (i.e. evenly) expressed across tissues. The red line is the trendline of a linear model. Source data are provided as a Source Data file.

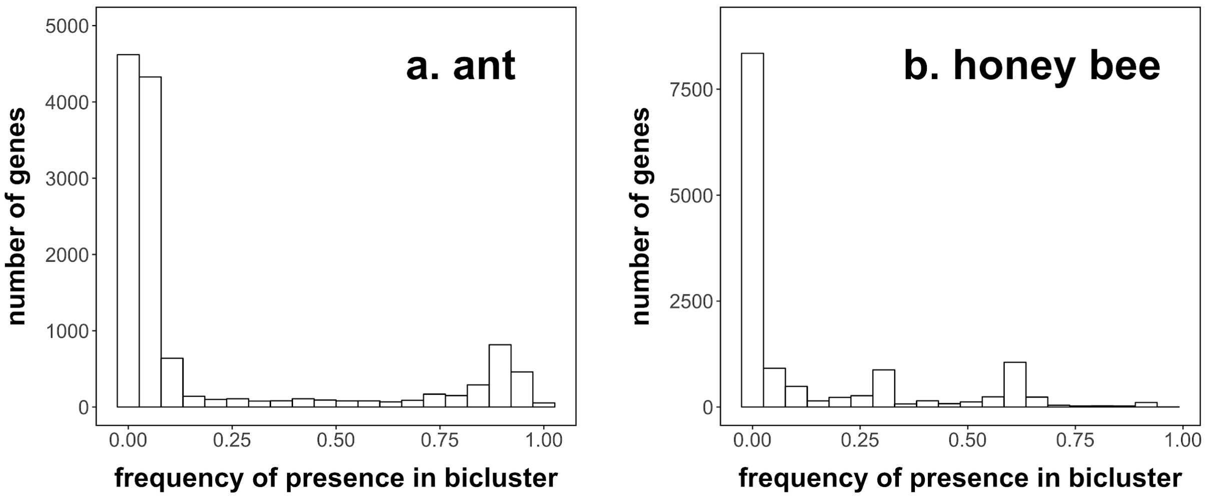

Supplementary Figure 14. Distributions of the frequencies by which genes were placed in biclusters associated with queen abdomens.
Histogram of the frequency with which genes were placed in the queen abdomen bicluster (out of 1000 runs). Plaid biclustering is a non-deterministic process, so different sets of genes can be present in each run. In ants (a), 1039 genes were present in >90% of queen abdomen biclusters and retained for further analysis. There are two peaks in the frequency distribution for honey bees (b). The lower frequency peak is made up of worker-associated genes (downregulated in queen abdomens) while the higher frequency peak (~60%) is made up of queen-associated genes. We retained genes with >60% frequency for further analysis. Source data are provided as a Source Data file.

Species	Stage	Tissue	Caste	Type	Replicates
ant	egg	whole body	N/A	N/A	6
ant	L1	whole body	N/A	N/A	6
ant	L2	whole body	queen	N/A	3
ant	L2	whole body	worker	N/A	3
ant	L3	whole body	queen	N/A	3
ant	L3	whole body	worker	N/A	3
ant	L4	whole body	queen	N/A	3
ant	L4	whole body	worker	N/A	3
ant	L5	whole body	queen	N/A	3
ant	L5	whole body	worker	N/A	3
ant	pupa	whole body	queen	N/A	3
ant	pupa	whole body	worker	N/A	3
ant	pupa	whole body	male	N/A	3
ant	adult	head	queen	virgin queen	3
ant	adult	thorax	queen	virgin queen	3
ant	adult	abdomen	queen	virgin queen	3
ant	adult	head	queen	mated queen	3
ant	adult	thorax	queen	mated queen	3
ant	adult	abdomen	queen	mated queen	3
ant	adult	head	worker	nurse	3
ant	adult	thorax	worker	nurse	3
ant	adult	abdomen	worker	nurse	3
ant	adult	head	worker	forager	3
ant	adult	thorax	worker	forager	3
ant	adult	abdomen	worker	forager	3
ant	adult	head	male	N/A	3
ant	adult	thorax	male	N/A	3
ant	adult	abdomen	male	N/A	3
honey bee	egg	whole body	N/A	N/A	3
honey bee	L1	whole body	N/A	N/A	3
honey bee	L2	whole body	queen	N/A	3
honey bee	L2	whole body	worker	N/A	3
honey bee	L3	whole body	queen	N/A	3
honey bee	L3	whole body	worker	N/A	3
honey bee	L4	whole body	queen	N/A	3
honey bee	L4	whole body	worker	N/A	3
honey bee	L5	whole body	queen	N/A	3
honey bee	L5	whole body	worker	N/A	4
honey bee	pupa	whole body	queen	N/A	5
honey bee	pupa	whole body	worker	N/A	3
honey bee	pupa	whole body	male	N/A	3
honey bee	adult	head	queen	virgin queen	3
honey bee	adult	thorax	queen	virgin queen	3
honey bee	adult	abdomen	queen	virgin queen	3
honey bee	adult	head	queen	mated queen	3
honey bee	adult	thorax	queen	mated queen	3
honey bee	adult	abdomen	queen	mated queen	3
honey bee	adult	head	worker	nurse	3
honey bee	adult	thorax	worker	nurse	3
honey bee	adult	abdomen	worker	nurse	3
honey bee	adult	head	worker	forager	3
honey bee	adult	thorax	worker	forager	3
honey bee	adult	abdomen	worker	forager	3
honey bee	adult	head	male	N/A	3
honey bee	adult	thorax	male	N/A	3
honey bee	adult	abdomen	male	N/A	3

Supplementary Table 1. Full listing of sample types and number of each sample collected. "L1" and "L2" refers to larvae of the first and second stage, etc. We began caste-specific sampling at stage two because caste is determined and regulated in *M. pharaonis* by the end of the first larval instar [1]. After the first larval instar in *M. pharaonis*, worker-destined larvae can be distinguished from reproductive-destined larvae, which include male-destined and queen-destined larvae [2]. As such, our "queen-destined" ant larvae samples likely contain some male-destined larvae, but the proportion is expected to be low, as the sex ratio is known to be heavily queen-biased [3]. Sex and caste are both known in *A. mellifera* larvae, as individuals are reared in separate cells [4].

stage/tissue	species	total DEGs	queen associated	worker associated
L2	ant	119	31	88
L3	ant	818	490	328
L4	ant	81	65	16
L5	ant	757	437	320
pupa	ant	290	88	202
head	ant	741	420	321
thorax	ant	1327	695	632
abdomen	ant	4395	2711	1684
larva_overall	ant	361	241	120
L2	honey bee	136	60	76
L3	honey bee	117	28	89
L4	honey bee	724	224	500
L5	honey bee	1009	540	469
pupa	honey bee	245	163	82
head	honey bee	1144	717	427
thorax	honey bee	1369	721	648
abdomen	honey bee	5352	2769	2583
larva_overall	honey bee	473	176	297

Supplementary Table 2. Number of differentially expressed genes (DEGs) between queens and workers for each comparison (FDR < 0.1). “L2”, “L3”, etc refer to the 2nd and 3rd larval stage, respectively, while “larva_overall” is the result of differential expression with caste as main effect across all larval samples. Differentially expressed genes are divided into “queen associated”, which exhibited higher expression in queens, and “worker associated”, which exhibited higher expression in workers. Differential expression analysis performed with N = 10804 (ant) and 11775 (honey bee) genes.

GO.ID	Term	P	stage/tissue
GO:0007265	Ras protein signal transduction	0.00026	larva
GO:0046578	regulation of Ras protein signal transduction	0.00029	larva
GO:0051056	regulation of small GTPase mediated signal transduction	0.00048	larva
GO:0007264	small GTPase mediated signal transduction	0.00071	larva
GO:0060628	regulation of ER to Golgi vesicle-mediated transport	0.00071	larva
GO:0006970	response to osmotic stress	0.00089	pupa
GO:0009651	response to salt stress	0.00287	pupa
GO:0019432	triglyceride biosynthetic process	0.00297	pupa
GO:0046460	neutral lipid biosynthetic process	0.00297	pupa
GO:0046463	acylglycerol biosynthetic process	0.00297	pupa
GO:0072525	pyridine-containing compound biosynthetic process	0.0018	head
GO:0001704	formation of primary germ layer	0.0042	head
GO:0002098	tRNA wobble uridine modification	0.0050	head
GO:0043086	negative regulation of catalytic activity	0.0071	head
GO:0010508	positive regulation of autophagy	0.0078	head
GO:0019362	pyridine nucleotide metabolic process	0.00021	thorax
GO:0046496	nicotinamide nucleotide metabolic process	0.00021	thorax
GO:0006733	oxidoreduction coenzyme metabolic process	0.00043	thorax
GO:0072524	pyridine-containing compound metabolic process	0.00054	thorax
GO:0006739	NADP metabolic process	0.00088	thorax
GO:0042445	hormone metabolic process	7.5e-05	abdomen
GO:0010817	regulation of hormone levels	0.00041	abdomen
GO:0042181	ketone biosynthetic process	0.00089	abdomen
GO:0042180	cellular ketone metabolic process	0.00094	abdomen
GO:0034754	cellular hormone metabolic process	0.00108	abdomen

Supplementary Table 3. Enriched gene ontology terms based on Gene Set Enrichment Analysis (GSEA) of differential expression between queens and workers in ants. P-value derived from Kolmogorov-Smirnov tests.

GO.ID	Term	P	stage/tissue
GO:0019722	calcium-mediated signaling	0.0010	larva
GO:0046113	nucleobase catabolic process	0.0021	larva
GO:0007411	axon guidance	0.0024	larva
GO:0061564	axon development	0.0027	larva
GO:0035039	male pronucleus assembly	0.0032	larva
GO:0008544	epidermis development	0.00053	pupa
GO:0008286	insulin receptor signaling pathway	0.00065	pupa
GO:0034599	cellular response to oxidative stress	0.00097	pupa
GO:0032869	cellular response to insulin stimulus	0.00106	pupa
GO:0071375	cellular response to peptide hormone stimulus	0.00106	pupa
GO:0008610	lipid biosynthetic process	0.00011	head
GO:0016070	RNA metabolic process	0.00018	head
GO:0030534	adult behavior	0.00028	head
GO:0008344	adult locomotory behavior	0.00031	head
GO:0007478	leg disc morphogenesis	0.00041	head
GO:0048747	muscle fiber development	0.0011	thorax
GO:0090254	cell elongation involved in imaginal disc-derived wing morphogenesis	0.0027	thorax
GO:0006457	protein folding	0.0045	thorax
GO:0071897	DNA biosynthetic process	0.0055	thorax
GO:0034063	stress granule assembly	0.0071	thorax
GO:0045887	positive regulation of synaptic growth at neuromuscular junction	0.00037	abdomen
GO:1904398	positive regulation of neuromuscular junction development	0.00037	abdomen
GO:0051965	positive regulation of synapse assembly	0.00116	abdomen
GO:0030490	maturation of SSU-rRNA	0.00126	abdomen
GO:0007436	larval salivary gland morphogenesis	0.00205	abdomen

Supplementary Table 4. Enriched gene ontology terms based on Gene Set Enrichment Analysis (GSEA) of differential expression between queens and workers in honey bees. P-value derived from Kolmogorov-Smirnov tests.

stage/tissue	species	total DEGs	nurse associated	forager associated
head	ant	405	314	91
thorax	ant	490	305	185
abdomen	ant	544	341	203
head	honey bee	927	404	523
thorax	honey bee	2519	1243	1276
abdomen	honey bee	2017	1007	1010

Supplementary Table 5. Number of differentially expressed genes (DEGs) between nurses and foragers for each comparison (FDR < 0.1). Differentially expressed genes are divided into "nurse associated", which exhibited higher expression in nurses, and "forager associated", which exhibited higher expression in foragers. Differential expression analysis performed with N = 10804 (ant) and 11775 (honey bee) genes.

GO.ID	Term	P	tissue
GO:0048284	organelle fusion	0.00021	head
GO:0006629	lipid metabolic process	0.00138	head
GO:0048580	regulation of post-embryonic development	0.00186	head
GO:0044255	cellular lipid metabolic process	0.00217	head
GO:0044801	single-organism membrane fusion	0.00226	head
GO:0032502	developmental process	0.00019	thorax
GO:0007525	somatic muscle development	0.00035	thorax
GO:0044767	single-organism developmental process	0.00041	thorax
GO:0007275	multicellular organism development	0.00041	thorax
GO:0090175	regulation of establishment of planar polarity	0.00043	thorax
GO:0000289	nuclear-transcribed mRNA poly(A) tail shortening	0.0017	abdomen
GO:0007006	mitochondrial membrane organization	0.0054	abdomen
GO:0042441	eye pigment metabolic process	0.0056	abdomen
GO:0043324	pigment metabolic process involved in developmental pigmentation	0.0056	abdomen
GO:0043474	pigment metabolic process involved in pigmentation	0.0056	abdomen

Supplementary Table 6. Enriched gene ontology terms based on Gene Set Enrichment Analysis (GSEA) of differential expression between nurses and foragers in ants. P-value derived from Kolmogorov-Smirnov tests.

GO.ID	Term	P	stage/tissue
GO:0019722	calcium-mediated signaling	0.0010	larva
GO:0046113	nucleobase catabolic process	0.0021	larva
GO:0007411	axon guidance	0.0024	larva
GO:0061564	axon development	0.0027	larva
GO:0035039	male pronucleus assembly	0.0032	larva
GO:0008544	epidermis development	0.00053	pupa
GO:0008286	insulin receptor signaling pathway	0.00065	pupa
GO:0034599	cellular response to oxidative stress	0.00097	pupa
GO:0032869	cellular response to insulin stimulus	0.00106	pupa
GO:0071375	cellular response to peptide hormone stimulus	0.00106	pupa
GO:0008610	lipid biosynthetic process	0.00011	head
GO:0016070	RNA metabolic process	0.00018	head
GO:0030534	adult behavior	0.00028	head
GO:0008344	adult locomotory behavior	0.00031	head
GO:0007478	leg disc morphogenesis	0.00041	head
GO:0048747	muscle fiber development	0.0011	thorax
GO:0090254	cell elongation involved in imaginal disc-derived wing morphogenesis	0.0027	thorax
GO:0006457	protein folding	0.0045	thorax
GO:0071897	DNA biosynthetic process	0.0055	thorax
GO:0034063	stress granule assembly	0.0071	thorax
GO:0045887	positive regulation of synaptic growth at neuromuscular junction	0.00037	abdomen
GO:1904398	positive regulation of neuromuscular junction development	0.00037	abdomen
GO:0051965	positive regulation of synapse assembly	0.00116	abdomen
GO:0030490	maturation of SSU-rRNA	0.00126	abdomen
GO:0007436	larval salivary gland morphogenesis	0.00205	abdomen

Supplementary Table 7. Enriched gene ontology terms based on Gene Set Enrichment Analysis (GSEA) of differential expression between nurses and foragers in honey bees. P-value derived from Kolmogorov-Smirnov tests.

Gene	logFC queen/worker	connectivity	SwissProt
LOC105837185	9.333	0.793	Leukocyte elastase inhibitor A
LOC105838268	8.610	0.790	Gephyrin
LOC105836111	8.147	0.784	RCC1 and BTB domain-containing protein 1
LOC105836023	7.962	0.772	Histone H2B
LOC105834654	7.258	0.797	Vitellogenin receptor
LOC105837528	6.871	0.848	Ankyrin-2
LOC105838623	6.713	0.855	Transcription factor SOX-14
LOC105829700	6.447	0.839	Maternal embryonic leucine zipper kinase
LOC105834441	6.307	0.864	Nuclear RNA export factor 1
LOC105830728	6.115	0.911	S-phase kinase-associated protein 2
LOC105837219	5.825	0.926	Rac GTPase-activating protein 1
LOC105840891	5.777	0.973	Acidic repeat-containing protein
LOC105838786	5.389	0.824	Spondin-1
LOC105837988	5.364	0.819	Multiple PDZ domain protein
LOC105830806	5.311	0.833	Insulin-degrading enzyme
LOC105836312	5.144	0.797	Serine protease nudel
LOC105836129	5.108	0.915	Rho GTPase-activating protein 19
LOC105834586	5.078	0.906	Putative bifunctional UDP-N-acetylglucosamine transferase and deubiquitinase ALG13
LOC105832223	5.058	0.874	E3 ubiquitin-protein ligase SIAH1
LOC105840292	4.974	0.785	Pre-mRNA-splicing factor RBM22
LOC105840093	4.833	0.897	ATP-dependent RNA helicase vasa isoform A
LOC105833898	4.812	0.925	Piwi-like protein 1
LOC105839662	4.676	0.966	G2/mitotic-specific cyclin-B3
LOC105828383	4.542	0.949	Histone RNA hairpin-binding protein
LOC105831777	4.326	0.815	Protein dispatched
LOC105835848	4.292	0.807	Glyoxylate reductase
LOC105838831	4.242	0.890	Broad-complex core protein isoform 6
LOC105832464	4.188	0.774	Zinc finger protein 800
LOC105837226	4.096	0.883	DNA repair and recombination protein RAD54-like (Fragment)
LOC105834656	4.077	0.805	Putative ATP-dependent RNA helicase me31b

Supplementary Table 8. Hub genes of the queen abdominal module in ants. Hub genes were defined as genes with intra-modular connectivity in at least the 90th percentile, and $\log_2$ fold-change (queen/worker) greater than 2.

Gene	logFC queen/worker	connectivity	SwissProt
LOC724752	11.662	0.746	E3 ubiquitin-protein ligase TRIM71
LOC410888	9.000	0.829	lachesin-like
LOC410684	7.405	0.754	homeobox protein OTX1 A
LOC100576333	6.493	0.738	nucleoredoxin-like
LOC725841	6.339	0.897	hyaluronan mediated motility receptor
LOC100577382	6.272	0.896	targeting protein for Xklp2 homolog
LOC551099	6.172	0.760	coiled-coil domain-containing protein 43
LOC724193	5.957	0.859	kinesin-like protein KIF18A
LOC726506	5.927	0.828	protein claret segregational
LOC725920	5.829	0.875	vitellogenin receptor
LOC100576828	5.826	0.843	protein maelstrom 2
LOC412031	5.811	0.930	S-phase kinase-associated protein 2
LOC102656846	5.705	0.942	cyclin-A2
LOC411529	5.531	0.861	maternal embryonic leucine zipper kinase-like
LOC410502	5.456	0.910	transformation/transcription domain-associated protein
LOC410015	5.270	0.778	protein LSM14 homolog A
LOC100578691	5.186	0.909	rhoGEF domain-containing protein gxcJ-like
LOC100578255	5.073	0.734	ras GTPase-activating-like protein IQGAP1
LOC552100	4.953	0.809	protein ovo
LOC100576908	4.883	0.917	polycomb protein Asx
LOC551871	4.808	0.912	P protein-like
LOC411970	4.546	0.767	G kinase-anchoring protein 1-like
LOC725606	4.498	0.758	serine protease gd
LOC409092	4.486	0.850	enhancer of mRNA-decapping protein 3
LOC409681	4.483	0.807	RWD domain-containing protein 1
LOC411809	4.460	0.780	enolase-phosphatase E1
LOC551773	4.320	0.876	serine/threonine-protein kinase VRK1-like
LOC413667	4.212	0.914	G2/mitotic-specific cyclin-B3
LOC409472	3.995	0.975	protein Smaug homolog 1
LOC552725	3.868	0.848	N-acetylglucosamine-1-phosphotransferase subunits alpha/beta

Supplementary Table 9. Hub genes of the queen abdominal module in honey bees. Hub genes were defined as genes with intra-modular connectivity in at least the 90th percentile, and $\log_2$ fold-change (queen/worker) greater than 2.

GO.ID	Term	P	test
GO:0010977	negative regulation of neuron projection development	0.00032	ant caste
GO:0014017	neuroblast fate commitment	0.00038	ant caste
GO:0045165	cell fate commitment	0.00041	ant caste
GO:0007400	neuroblast fate determination	0.00085	ant caste
GO:0010771	negative regulation of cell morphogenesis involved in differentiation	0.00122	ant caste
GO:0060322	head development	2.3e-07	ant behavior
GO:0007420	brain development	3.6e-07	ant behavior
GO:0016319	mushroom body development	1.6e-05	ant behavior
GO:0045165	cell fate commitment	2.1e-05	ant behavior
GO:0007417	central nervous system development	4.2e-05	ant behavior
GO:0006650	glycerophospholipid metabolic process	0.00046	bee caste
GO:0051231	spindle elongation	0.00098	bee caste
GO:0046488	phosphatidylinositol metabolic process	0.00134	bee caste
GO:0035050	embryonic heart tube development	0.00194	bee caste
GO:0000022	mitotic spindle elongation	0.00222	bee caste
GO:0035295	tube development	0.00020	bee behavior
GO:0002009	morphogenesis of an epithelium	0.00029	bee behavior
GO:0035239	tube morphogenesis	0.00032	bee behavior
GO:0048729	tissue morphogenesis	0.00061	bee behavior
GO:0060562	epithelial tube morphogenesis	0.00123	bee behavior

Supplementary Table 10. Enriched gene ontology terms based on overall caste or behavior bias in ants and honey bees. GO terms are derived from *D. melanogaster* orthologs. P-value is from gene set enrichment analysis (Kolmogorov–Smirnov test)

species	comparison	abdomen included?	variable tested	Spearman rho	P-value
ant	caste	yes	connectivity	-0.162	3.92e-33
ant	caste	no	connectivity	-0.282	1.38e-99
ant	caste	yes	dN/dS	0.151	6.07e-29
ant	caste	no	dN/dS	0.130	8.51e-22
ant	caste	yes	evolutionary age	0.159	5.19e-32
ant	caste	no	evolutionary age	0.161	5.96e-33
honey bee	caste	yes	connectivity	0.003	8.13e-01
honey bee	caste	no	connectivity	-0.132	4.61e-20
honey bee	caste	yes	dN/dS	0.155	3.15e-27
honey bee	caste	no	dN/dS	0.169	4.27e-32
honey bee	caste	yes	evolutionary age	0.166	3.72e-31
honey bee	caste	no	evolutionary age	0.154	7.95e-27
honey bee	caste	yes	tau	0.409	1.33e-193
honey bee	caste	no	tau	0.397	1.14e-180
ant	behavior	yes	connectivity	-0.157	4.29e-31
ant	behavior	no	connectivity	-0.119	1.24e-18
ant	behavior	yes	dN/dS	0.043	1.75e-03
ant	behavior	no	dN/dS	0.020	1.38e-01
ant	behavior	yes	evolutionary age	0.021	1.25e-01
ant	behavior	no	evolutionary age	0.014	3.02e-01
honey bee	behavior	yes	connectivity	-0.162	9.22e-30
honey bee	behavior	no	connectivity	-0.137	1.49e-21
honey bee	behavior	yes	dN/dS	0.174	7.02e-34
honey bee	behavior	no	dN/dS	0.150	1.83e-25
honey bee	behavior	yes	evolutionary age	0.161	3.49e-29
honey bee	behavior	no	evolutionary age	0.126	1.95e-18
honey bee	behavior	yes	tau	0.328	9.55e-121
honey bee	behavior	no	tau	0.281	5.79e-88

Supplementary Table 11. Partial correlation between connectivity, evolutionary rate (dN/dS), evolutionary age (phylostrata), and tissue-specificity (tau) and caste or behavior bias while accounting for expression. Analysis was performed separately for each species and comparison (i.e. separately for caste bias and behavior bias), as well as while including or excluding abdomen in calculations of caste bias and expression. Connectivity is total connectivity measured across all samples and genes. Phylostrata is a measure of estimated evolutionary age, with higher values indicating younger genes. Tau is the degree to which genes exhibit tissue-specific expression across 12 honey bee tissues (results presented only for honey bees). N = 10520 genes (ants), 10011 genes (honey bees). To estimate a measure of expression analogous to overall bias, we calculated the Euclidean distance of $\log_{10}$ counts-per-million at each stage/tissue tested.

Supplementary Table 12. List of species used for phylostratigraphy analysis, with the NCBI Taxonomy ID.

Species	NCBI Taxonomy ID
Acromyrmex echinatior	103372
Atta cephalotes	12957
Atta colombica	520822
Camponotus floridanus	104421
Cardiocondyla obscurior	286306
Monomorium pharaonis	307658
Linepithema humile	83485
Lasius niger	67767
Harpegnathos saltator	610380
Dinoponera quadriceps	609295
Cyphomyrmex costatus	456900
Ooceraea biroi	2015173
Pogonomyrmex barbatus	144034
Pseudomyrmex gracilis	219809
Solenopsis invicta	13686
Trachymyrmex septentrionalis	34720
Trachymyrmex cornetzi	471704
Trachymyrmex zeteki	64791
Vollenhovia emeryi	411798
Wasmannia auropunctata	64793
Temnothorax curvispinosus	300111
Apis cerana	7461
Apis dorsata	7462
Apis florea	7463
Apis mellifera	7460
Bombus impatiens	132113
Bombus terrestris	30195
Melipona quadrifasciata	166423
Eufriesea mexicana	516756
Ceratina calcarata	156304
Megachile rotundata	143995
Habropoda laboriosa	597456
Dufourea novaeangliae	178035
Polistes canadensis	91411
Polistes dominula	743375
Ceratosolen solmsi	142686
Ceratosolen solmsi marchali	326594
Copidosoma floridanum	29053
Fopius arisanus	64838
Microplitis demolitor	69319
Nasonia vitripennis	7425
Trichogramma pretiosum	7493
Trichomalopsis sarcophagae	543379
Diachasma alloeum	454923
Orussus abietinus	222816
Athalia rosae	37344
Cephus cinctus	211228
Neodiprion lecontei	441921
Pediculus humanus	121225
Drosophila melanogaster	7227
Aedes aegypti	7159
Bombyx mori	7091
Papilio machaon	76193
Anopheles gambiae	7165
Onthophagus taurus	166361
Tribolium castaneum	7070
Acyrthosiphon pisum	7029
Zootermopsis nevadensis	136037
Caenorhabditis elegans	6239
Hydra vulgaris	6087
Strongylocentrotus purpuratus	7668
Lottia gigantea	225164
Helobdella robusta	6412
Mus musculus	10090
Homo sapiens	9606
Xenopus tropicalis	8364
Latimeria chalumnae	7897
Danio rerio	7955

Supplementary References

1. Warner, M. R., Kovaka, K. & Linksvayer, T. A. Late-instar ant worker larvae play a prominent role in colony-level caste regulation. Insectes Soc. 63, 575–583 (2016).
2. Edwards, J. P. Caste regulation in the pharaoh's ant *Monomorium pharaonis*: the influence of queens on the production of new sexual forms. *Physiol. Entomol.* **12**, 31–39 (1987).
3. Schmidt, A. M., Linksvayer, T. A., Boomsma, J. J. & Pedersen, J. S. Queen–worker caste ratio depends on colony size in the pharaoh ant (Monomorium pharaonis). Insectes Soc. 58, 139–144 (2011).
4. Wilson, E. O. The Insect Societies. (Harvard University Press, 1971).
