# Supplementary material for: Convergent eusocial evolution is based on a shared reproductive groundplan plus lineage-specific plastic genes
Source: Nat Commun. 2019 Jun 14;10:2651. doi: 10.1038/s41467-019-10546-w (PMC6570765; doi:10.1038/s41467-019-10546-w)
Supplement: Supplementary file 3 — Reporting Summary [file 41467_2019_10546_MOESM3_ESM.pdf]

nature research

Corresponding author(s): *Double-blind peer review submissions: write DBPR and your manuscript number here instead of author names.*

Last updated by author(s): *YYYY-MM-DD*

Reporting Summary

Nature Research wishes to improve the reproducibility of the work that we publish. This form provides structure for consistency and transparency in reporting. For further information on Nature Research policies, see Authors & Referees and the Editorial Policy Checklist .

Statistics

For all statistical analyses, confirm that the following items are present in the figure legend, table legend, main text, or Methods section.

n/a	Confirmed	
☐	☒	The exact sample size (n) for each experimental group/condition, given as a discrete number and unit of measurement
☐	☒	A statement on whether measurements were taken from distinct samples or whether the same sample was measured repeatedly
☐	☒	The statistical test(s) used AND whether they are one- or two-sided<br>*Only common tests should be described solely by name; describe more complex techniques in the Methods section.*
☐	☒	A description of all covariates tested
☐	☒	A description of any assumptions or corrections, such as tests of normality and adjustment for multiple comparisons
☐	☒	A full description of the statistical parameters including central tendency (e.g. means) or other basic estimates (e.g. regression coefficient) AND variation (e.g. standard deviation) or associated estimates of uncertainty (e.g. confidence intervals)
☐	☒	For null hypothesis testing, the test statistic (e.g. F, t, r) with confidence intervals, effect sizes, degrees of freedom and P value noted<br>*Give P values as exact values whenever suitable.*
☐	☒	For Bayesian analysis, information on the choice of priors and Markov chain Monte Carlo settings
☐	☒	For hierarchical and complex designs, identification of the appropriate level for tests and full reporting of outcomes
☐	☒	Estimates of effect sizes (e.g. Cohen's d, Pearson's r), indicating how they were calculated

Our web collection on statistics for biologists contains articles on many of the points above.

Software and code

Policy information about availability of computer code

Data collection	All source code will be available on github upon article publication
Data analysis	All source code will be available on github upon article publication

For manuscripts utilizing custom algorithms or software that are central to the research but not yet described in published literature, software must be made available to editors/reviewers. We strongly encourage code deposition in a community repository (e.g. GitHub). See the Nature Research guidelines for submitting code & software for further information.

Data

Policy information about availability of data

All manuscripts must include a data availability statement. This statement should provide the following information, where applicable:

- Accession codes, unique identifiers, or web links for publicly available datasets
- A list of figures that have associated raw data
- A description of any restrictions on data availability

Raw data is uploaded to SRA. Processed data will be available on Github.

Field-specific reporting

Please select the one below that is the best fit for your research. If you are not sure, read the appropriate sections before making your selection.

☐ Life sciences ☐ Behavioural & social sciences ☒ Ecological, evolutionary & environmental sciences

For a reference copy of the document with all sections, see nature.com/documents/nr-reporting-summary-flat.pdf

Ecological, evolutionary & environmental sciences study design

All studies must disclose on these points even when the disclosure is negative.

Study description	We sampled individuals across caste development and across three adult tissues in pharaoh ants and honey bees (N = 177 RNA-seq samples total). We identified genes commonly associated with caste, and found that genes frequently associated with caste tended to be evolutionarily young, plastically expressed, and rapidly evolving.
Research sample	Pools of individuals across caste development in Apis mellifera and Monomorium pharaonis. Includes queen- and worker-destined larvae as well as queen, male, and worker pupae.
Sampling strategy	We collected three replicates for each caste/stage/tissue combination. This number was chosen to enable rigorous differential expression analysis within cost constraints.
Data collection	MRW and MJH collected individuals and LQ performed all RNA extractions and sequencing.
Timing and spatial scale	All samples were collected from laboratory colonies in summer 2016.
Data exclusions	Lowly expressed genes (TPM < 1 in all sample types) were excluded from analysis.
Reproducibility	No replicate experiments were performed.
Randomization	Organisms were not randomized, but sampled from separate replicate colonies.
Blinding	Blinding was not relevant as behavior was not assessed other than to collect worker nurses and foragers.

Did the study involve field work? ☐ Yes ☒ No

Reporting for specific materials, systems and methods

We require information from authors about some types of materials, experimental systems and methods used in many studies. Here, indicate whether each material, system or method listed is relevant to your study. If you are not sure if a list item applies to your research, read the appropriate section before selecting a response.

Materials & experimental systems

n/a	Involved in the study	
☒	☐	Antibodies
☒	☐	Eukaryotic cell lines
☒	☐	Palaeontology
☐	☒	Animals and other organisms
☒	☐	Human research participants
☒	☐	Clinical data

Methods

n/a	Involved in the study	
☒	☐	ChIP-seq
☒	☐	Flow cytometry
☒	☐	MRI-based neuroimaging

Animals and other organisms

Policy information about studies involving animals; ARRIVE guidelines recommended for reporting animal research

Laboratory animals	Monomorium pharaonis and Apis mellifera, various strains
Wild animals	study did not involve wild animals
Field-collected samples	study did not involve field-collected samples
Ethics oversight	No ethical approval was required as studied solely invertebrates.

Note that full information on the approval of the study protocol must also be provided in the manuscript.
